# Physiological and psychological effects of weight loss-induced stress before a competition in senior wrestlers

**DOI:** 10.3389/fpsyg.2025.1568284

**Published:** 2025-04-02

**Authors:** Bilal Okudan, Ozkan Isik, Zeynep Akyurek, Omur Fatih Karakullukcu, Laurentiu-Gabriel Talaghir, Liliana Nanu

**Affiliations:** ^1^Independent Researcher, Ankara, Türkiye; ^2^Faculty of Sport Sciences, Balikesir University, Balıkesir, Türkiye; ^3^Institute of Health Sciences, Balikesir University, Balıkesir, Türkiye; ^4^Ministry of National Education, Ankara, Türkiye; ^5^Faculty of Physical Education and Sport, Dunarea de Jos University of Galati, Galati, Romania

**Keywords:** dehydration, food and fluid restriction, stress, rapid weight loss, wrestling

## Abstract

**Background:**

It is known that stress occurring through/against a phenomenon will have physiological and psychological effects on the human organism. Therefore, this research aimed to determine the physiological and psychological effects of weight loss-induced stress before a competition in senior wrestlers.

**Methods:**

This cross-sectional study used a purposeful sampling method to select participants. Two hundred and forty-three wrestlers participated in the study voluntarily. The perceived stress scale and athlete weight loss methodology and effects scale were used to determine the physiological and psychological effects of weight loss-induced stress. Independent samples t-test, One-way ANOVA, Pearson correlation analysis, and linear regression analysis were used to analyze normally distributed data.

**Results:**

There was no statistical difference in the wrestlers’ body weight loss percentages, stress levels, and weight loss methods and effects sub-dimensions according to their gender and wrestling styles (*p* > 0.05). Despite this, there was a statistical difference in wrestlers’ stress levels, ergogenic aids sub-dimension from weight loss methods, and psychological effect levels according to body weight loss percentages (*p* < 0.05). Additionally, there was a positive relationship between the body weight loss percentages of wrestlers with their stress levels (*r*: 0.461) and psychological effects (*r*: 0.240). Furthermore, there was a positive relationship between the stress levels of wrestlers with the average physiological (*r*: 0.298) and psychological (*r*: 0.508) effects. According to these results, it was determined that as the body weight loss percentages of wrestlers increased, their stress levels and the psychological effects they were exposed to would increase. It was also determined that as the stress level of wrestlers increased, the physiological and psychological effects they were exposed to would increase the weight loss-induced stress levels of wrestlers positively predicted their exposure to physiological and psychological effects at 8.5% (Adj. *R*^2^ = 0.085) and 25.8% (Adj. *R*^2^ = 0.258), respectively.

**Conclusion:**

It was determined that as the percentage of body weight loss increases in wrestlers, the stress level will also increase and the stress level increases, the physiological and psychological effects will also increase. It is thought that weight loss-induced stress has both physiological and psychological effects on wrestlers and may reduce their athletic performance.

## 1 Introduction

Wrestling is one of the earliest combat sports humans have known. Since ancient times, wrestling has been widely taught to humanity and used to train soldiers (Isik and Gumus, 2018). Wrestling is a combat sport that is performed under certain rules and basic and combined motor abilities, as well as some sportive forms are used, and it includes both sociological and psychological factors (Isik et al., 2013). Today, wrestling still appears as the sports branch representing the most medals among combat sports in the Olympic Games in Paris 2024 (Seker et al., 2024). One of the most important rules in wrestling is that wrestlers compete according to their gender and body weight to prevent unfair competition (Kim and Park, 2021). For this reason, there are three different wrestling styles such as Greco-Roman (GR), Free-Style (FS), and women’s wrestling (WW) in the modern Olympics (Roklicer et al., 2022).

Previous studies have reported that most wrestlers perform Rapid Weight Loss (RWL) before a competition (Cicioglu et al., 2017; Donmez et al., 2025; Isik and Cicioglu, 2016; Isik et al., 2018; Roklicer et al., 2022; Slačanac et al., 2021). The basis of this RWL is the idea that wrestlers will gain an advantage by competing against weaker or smaller opponents (Artioli et al., 2016; Khodaee et al., 2015; Yalcin et al., 2019). The disruption in the body’s water balance due to the water lost from the body during the RWL process manifests itself as an abnormality (hypernatremia or hyponatremia) in the serum sodium concentration (Adrogué and Madias, 2000). Increased plasma tonicity (hypernatremia) causes disruption of homeostasis in the body and thus creates physical and psychological stress in the body. Stress, broadly defined as a real or perceived threat to homeostasis, activates neural circuits that alter the body’s physiology and behavior to ensure survival and well-being. Physical stressors are real threats that create signals within the internal environment that communicate deviations from homeostasis to the brain. In contrast, perceived threats or psychogenic stressors create signals that arise within the brain itself as it interprets stimuli in the external environment as potential insults. Although from different origins, physical and psychogenic stressors activate hypothalamic circuitry mediating reactive responses (Krause et al., 2011). If the weight loss-induced physical and psychological stressors are not eliminated to ensure hemostasis, the physical and psychological effects on wrestlers continue during the weight loss process.

There are many methods for RWL, such as diet (food and fluid restriction), dehydration (use of sauna, jogging in the raincoat, and spitting, etc.), and ergogenic aids (laxatives, diet pills, and diuretic pills, etc.), and wrestlers frequently use one or more of these methods for RWL (Castor-Praga et al., 2021; Seyhan, 2018; Yagmur et al., 2019b). Jogging with a raincoat and/or using a sauna during/after training for weight loss in wrestlers accelerates fluid excretion from the body through sweating, and this decrease in body water increases physiological stress in their body. Furthermore, food and fluid restrictions in addition to intensive workouts can create a negative mood in wrestlers and cause them to be affected psychologically (Karninčić et al., 2016). In a study conducted on wrestlers, Isik et al. (2018) reported that wrestlers who lost weight may be exposed to hyperosmolar pressure because of increased sodium levels due to decreased fluid levels in the body and may show hypernatremic responses (thirst, increased urine concentration, muscle cramps, dry skin and mouth, confusion, seizures or coma, etc.). RWL, with all these methods can affect wrestlers physiologically and expose them to many negative psychological effects. RWL has been reported to cause psychological effects in athletes, such as decreased short-term memory, vigor, concentration, and self-esteem, as well as increased confusion, anger, fatigue, depression, and isolation, all of which can hamper competitive performance (Franchini et al., 2012).

In sum, weight loss using various methods before a competition can increase wrestlers’ stress levels. For this reason, it is important to predict to what extent increased stress due to weight loss will affect wrestlers physiologically and psychologically. In this context, the primary purpose of this study was to determine the physiological and psychological effects of weight loss-induced stress before a competition in senior wrestlers. The secondary purpose of this study was to determine (a) whether there was a difference between senior wrestlers’ body weight loss percentages and weight loss methods and effects according to their gender and wrestling style and (b) whether there was a difference between senior wrestlers’ stress levels and weight loss methods and effects according to their gender and wrestling style.

## 2 Methods

### 2.1 Research design and participants

A purposeful sampling method to select participants was used in this cross-sectional study. The sample size’s power was calculated with G*Power software version 3.1.9.2 to generalize this study’s results. The number of participants required to expect a medium effect size (*f* = 0.30) from the research results was determined as 210 (*α* = 0.05; 1-*β* = 0.95). Two hundred fifty-two active wrestlers were initially included in the study voluntarily. Nine wrestlers were excluded because they did not lose weight. Finally, the study sample was expanded to 243 wrestlers ($\bar{Χ}$_Age_: 22.41 ± 3.33 years) and the current sample size shows that the results can be generalized.

### 2.2 Data collection tools

In the study, data was obtained from wrestlers voluntarily using the online survey method via Google Forms. In addition to the personal information form, the athlete weight loss methodology and effects scale, and perceived stress scale were used as measurement tools.

#### 2.2.1 Personal information form

The personal information form included questionaries about the wrestlers’ age, gender, wrestling style, normal body weight (before starting weight loss), and weight categories for competition. The percentage of body weight loss performed by wrestlers for a competition was calculated using the formula: Percentage of Body Weight Loss = [(Normal Body Weight − Weight Category for Competition)/Normal Body Weight] × 100 (Isik and Cicioglu, 2016; Isik et al., 2018). Moreover, the percentage of body weight loss of wrestlers was turned into a categorical variable according to the weight loss classification of Casa et al. (2000). According to Casa et al. (2000), ±1% of body weight loss is well hydrated, >1–3% is minimal dehydration, >3–5% is significant dehydration, and > 5% is serious dehydration.

#### 2.2.2 Athlete weight loss methodology and effects scale

To determine the weight loss methods of wrestlers and the possible effects of these methods, the Athlete weight loss methodology and effects scale developed by Yarar et al. (2016) was used. The Athlete weight loss methodology and effects scale was a five-point Likert-type (1 = Never, 2 = Rarely, 3 = Sometimes, 4 = Frequently, 5 = Always) scale consisting of 19 questions divided into five sub-dimensions. The sub-dimensions for weight loss methodology were diet (3 items), fluid loss (3 items), and ergogenic aids (3 items), while the sub-dimensions of weight loss effects were physiological effect (5 items) and psychological effect (5 items). The reliability coefficient (Cronbach’s alpha) for the original Athlete weight loss methodology and effects scale was 0.74. In this study, Cronbach’s alpha coefficient for Athlete weight loss methodology and effects scale was determined as 0.83. This result shows that the Athlete weight loss methodology and effects scale was reliable for wrestlers in this study sample.

#### 2.2.3 Perceived stress scale

To determine the stress levels perceived by wrestlers during weight loss, the Perceived Stress Scale, developed by Cohen et al. (1983) and adapted to Turkish by Eskin et al. (2013), was used. The Perceived Stress Scale, consisting of a total of 14 items and a single dimension, was designed to measure how stressful a person perceives certain situations in their life. Wrestlers rated each item on a 5-point Likert-type scale ranging from “Never (0)” to “Very often (4).” The 7 items with positive expressions were reversed and the scores to be obtained from the Perceived Stress Scale ranged from 0 to 56. High scores indicated that the person’s perceived stress level was high. The reliability coefficient (Cronbach’s alpha) for the original perceived stress scale was 0.84. In this study, Cronbach’s alpha coefficient for the perceived stress scale was determined as 0.91. This result shows that the perceived stress scale was reliable for wrestlers who perform weight loss.

### 2.3 Ethical approval

This study was ethically approved by the Balıkesir University Health Sciences Non-invasive Research Ethics Committee, with decision number 2025/23. In addition, informed consent was obtained from all participants before answering the questions.

### 2.4 Statistical analysis

In addition to descriptive statistics (i.e., percent, frequency, mean, and standard deviation), the Kolmogorov–Smirnov test and Skewness and Kurtosis values were checked for the normality test. As a result of the normality test of the obtained data, it was determined that the Kolmogorov–Smirnov test values for obtained data *p* > 0.05 and Skewness and Kurtosis values of the data varied between −2, ..., +2. These values obtained show that the data were suitable for normal distribution (George and Mallery, 2019). For this reason, parametric test techniques were used in the analysis of the obtained data. In the analysis of normally distributed data, the independent samples t-test was used for comparison according to gender. One-way ANOVA was used to compare wrestling styles and the percentages of body weight loss. The LSD post-hoc test was applied to determine the source of the difference between the percentage of body weight loss classifications. A Pearson correlation analysis was used to determine the relationship between the sub-dimensions of weight loss methods and effects scale, stress, and the percentage of body weight loss. Regression analysis was used to determine the physiological and psychological effects of weight loss-induced stress. Significance was set at *p* < 0.05.

## 3 Results

Of the wrestlers in the study, 44.9% were female and 55.1% were male. Additionally, 35% were FS, 20.2% were GR, and 44.9% were WW competitors. When wrestlers were classified according to their body weight loss percentages, there were no well-hydrated wrestlers and 18.1% were minimally dehydrated, 37.0% were significantly dehydrated, and 44.9% were seriously dehydrated (Table 1).

**Table 1:** Distribution of demographic characteristics of wrestlers.

Demographic characteristics	Categories	*f*	%
Gender	Women	109	44.9
	Men	134	55.1
Wrestling Styles	FS	85	35.0
	GR	49	20.2
	WW	109	44.9
Percentage of Body Weight Loss (%kg)	Well-hydrated	–	–
	Minimal dehydration	44	18.1
	Significant dehydration	90	37.0
	Serious dehydration	109	44.9

When the wrestlers’ body weight loss percentages, stress levels, and weight loss methods and effects sub-dimensions were compared according to gender (Table 2) and wrestling styles (Table 3), it was determined that there were no statistically significant differences for stress levels and weight loss methods and effects sub-dimensions (*p* > 0.05).

**Table 2:** Comparison of stress levels and weight loss methods and effects sub-dimensions in wrestlers according to gender.

Variables		Gender	*n*	Mean ± Std. deviation	*t*	*p*
Percentage of Body Weight Loss (%kg)		Women	109	5.59 ± 2.75	1.006	0.315
		Men	134	5.24 ± 2.59		
Stress		Women	109	28.67 ± 8.85	1.366	0.173
		Men	134	27.21 ± 7.80		
Sub-Dimensions of Weight Loss Methods and Effects Scale	Diet	Women	109	2.72 ± 1.03	0.837	0.403
		Men	134	2.62 ± 0.89		
	Dehydration	Women	109	2.33 ± 0.60	−0.793	0.428
		Men	134	2.40 ± 0.66		
	Ergogenic aids	Women	109	1.47 ± 0.77	0.039	0.969
		Men	134	1.47 ± 0.75		
	Physiological effect	Women	109	1.99 ± 0.73	0.620	0.536
		Men	134	1.93 ± 0.69		
	Psychological effect	Women	109	2.30 ± 0.67	0.149	0.881
		Men	134	2.29 ± 0.78		

*p > 0.05.*

**Table 3:** Comparison of stress levels and weight loss methods and effects sub-dimensions in wrestlers according to wrestling styles.

Variables		Wrestling styles	*n*	Mean ± Std. deviation	*F*	*p*
Percentage of Body Weight Loss (%kg)		FS	85	5.23 ± 2.57	0.508	0.602
		GR	49	5.27 ± 2.64		
		WW	109	5.59 ± 2.75		
Stress		FS	85	27.34 ± 7.99	0.959	0.385
		GR	49	26.98 ± 7.55		
		WW	109	28.67 ± 8.85		
Sub-Dimensions of Weight Loss Methods and Effects Scale	Diet	FS	85	2.63 ± 0.91	0.360	0.698
		GR	49	2.61 ± 0.86		
		WW	109	2.72 ± 1.03		
	Dehydration	FS	85	2.35 ± 0.67	1.014	0.364
		GR	49	2.48 ± 0.62		
		WW	109	2.33 ± 0.60		
	Ergogenic aids	FS	85	1.44 ± 0.79	0.245	0.783
		GR	49	1.53 ± 0.68		
		WW	109	1.47 ± 0.77		
	Physiological effect	FS	85	1.92 ± 0.68	0.239	0.787
		GR	49	1.96 ± 0.70		
		WW	109	1.99 ± 0.73		
	Psychological effect	FS	85	2.28 ± 0.86	0.020	0.980
		GR	49	2.30 ± 0.62		
		WW	109	2.30 ± 0.67		

*p > 0.05.*

When the wrestlers’ sub-dimensions of the weight loss methods and effects scale were compared according to body weight loss percentages; it was determined that there was no statistically significant difference in the diet, dehydration, and physiological effect sub-dimensions (p > 0.05), whereas there was a statistically significant difference in the ergogenic aids and psychological effects sub-dimensions and stress levels (*p* < 0.05). According to these results, there was a statistically significant difference between the stress levels of all groups in terms of body weight loss percentage. In the ergogenic aids and psychological effects sub-dimensions, it was determined that wrestlers in the serious dehydration group had higher averages than wrestlers in other groups (Table 4).

**Table 4:** Comparison of stress levels and weight loss methods and effects sub-dimensions in wrestlers according to classification of percentage of body weight loss.

Variables		Percentage of body weight loss (%kg)	*n*	Mean ± Std. deviation	*F*	*p*
Stress		Minimal Dehydration	44	18.98 ± 5.40^†^	60.435	0.001*
		Significant Dehydration	90	26.97 ± 8.01^‡^		
		Serious Dehydration	109	32.19 ± 6.19^#^		
Sub-Dimensions of Weight Loss Methods and Effects Scale	Diet	Minimal Dehydration	44	2.56 ± 1.03	0.931	0.396
		Significant Dehydration	90	2.61 ± 0.85		
		Serious Dehydration	109	2.76 ± 1.00		
	Dehydration	Minimal Dehydration	44	2.23 ± 0.58	2.644	0.073
		Significant Dehydration	90	2.32 ± 0.60		
		Serious Dehydration	109	2.46 ± 0.67		
	Ergogenic aids	Minimal Dehydration	44	1.30 ± 0.71^‡^	5.794	0.003*
		Significant Dehydration	90	1.34 ± 0.65^‡^		
		Serious Dehydration	109	1.65 ± 0.82^#^		
	Physiological effect	Minimal Dehydration	44	1.80 ± 0.76	1.800	0.168
		Significant Dehydration	90	1.95 ± 0.71		
		Serious Dehydration	109	2.03 ± 0.67		
	Psychological effect	Minimal Dehydration	44	1.98 ± 0.67^‡^	9.472	0.001*
		Significant Dehydration	90	2.21 ± 0.80^‡^		
		Serious Dehydration	109	2.49 ± 0.63^#^		

*p < 0.05; #, ‡, †: Different figures show statistical differences between groups.

When the relationship between the percentages of body weight loss, stress levels, and physiological and psychological effects of wrestlers was examined, it was determined that there was a positive relationship between the percentage of body weight loss with stress levels (*r*: 0.461) and psychological (0.240) effects. It was also determined that there was a positive relationship between the stress levels of wrestlers with the average physiological (*r*: 0.298) and psychological (*r*: 0.508) effects. According to these results, it was determined that as the percentages of body weight loss of wrestlers increased, their stress levels and the psychological effects they were exposed to would increase. Furthermore, it was determined that as the stress level of wrestlers increased, the physiological and psychological effects they were exposed to would increase (Table 5).

**Table 5:** Relationship between body weight loss percentage with stress levels and weight loss effects in wrestlers.

Variables		Percentage of body weight loss (%kg)	Stress	Physiological effects	Psychological effects
Percentage of body weight loss (%kg)	*r*	1			
	*p*				
Stress	*r*	0.461	1		
	*p*	0.001*			
Physiological effects	*r*	0.054	0.298	1	
	*p*	0.406	0.001*		
Psychological effects	*r*	0.240	0.508	0.485	1
	*p*	0.001*	0.001*	0.001*	

*p < 0.05.

Linear regression analysis was used to determine the effect of weight loss-induced stress levels on the physiological and psychological effects of wrestlers. It was determined that the weight loss-induced stress levels of wrestlers positively predicted their exposure to physiological (*β* = 0.298; *t* = 4.844; *p* = 0.001) and psychological (*β* = 0.508; *t* = 9.149; *p* = 0.001) effects at the levels of 8.5% (Adj. *R*^2^ = 0.085) and 25.8% (Adj. *R*^2^ = 0.258), respectively (Table 6).

**Table 6:** Physiological and psychological effects of weight loss-induced stress in wrestlers.

Independent variables	*β*	*t*	*p*	*F*	Adj. *R*^2^
(Constant)		8.261	0.001*	23.460	0.085
Stress	0.298	4.844	0.001*		

Dependent variables Physiological effects					Method: Enter
(Constant)		7.410	0.001*	83.701	0.258
Stress	0.508	9.149	0.001*		

Dependent variables Psychological effects		Method: Enter

*p < 0.05.

## 4 Discussion

This research was carried out to reveal to what extent weight loss-induced stress affects senior wrestlers physiologically and psychologically. This study included 109 women and 134 men (49 wrestlers in GR and 85 wrestlers in FS) wrestlers and found that 44.9% of them were exposed to serious dehydration, losing more than 5% of their body weight (Table 1). While many studies have well-documented the negative effects of RWL on wrestlers (Kukić et al., 2021; Lukic-Sarkanovic et al., 2024; Milovančev et al., 2023; Seker et al., 2024; Trivic et al., 2023; Yagmur et al., 2019a), the reasons why competitive wrestlers still perform high levels of RWL are not fully understood.

The percentages of body weight loss, stress, and weight loss methods, and the physiological and psychological effects of weight loss methods were not different according to the wrestlers’ gender (Table 2) and wrestling styles (Table 3). Our results show that the percentages of body weight loss, stress, and weight loss methods used by women and men wrestlers were similar and that they were exposed to similar physiological and psychological effects before a competition in senior wrestlers. When the studies in the literature were examined, it was reported that there was no difference in percentages of body weight loss in both judoka (Artioli et al., 2010) and wrestlers (Seker et al., 2024). Furthermore, since wrestling styles were also directly related to wrestlers’ gender, there was also no difference between FS, GR, and WW in terms of body weight loss percentage.

Various classification methods have been employed to investigate the effects of weight loss in wrestlers, with a particular focus on its physiological and psychological consequences. While some researchers have assessed weight loss in absolute terms (kg) (Coufalova et al., 2013; Figlioli et al., 2021; Isik et al., 2013), others argue that expressing weight loss as a percentage of body weight (%) provides a more accurate and standardized measure (Ceylan and Balci, 2023; Pettersson and Berg, 2014). In another study, Casa et al. (2000) categorized weight loss percentages and highlighted that serious dehydration, defined as a loss exceeding 5% of body weight, induces substantial physiological and psychological impairments. In our study, it was determined that there was a statistically significant difference in stress levels, ergogenic aids, one of the weight loss methods, and psychological factors, one of the weight loss effects of wrestlers according to body weight loss percentages. According to these results, it was observed that the stress levels of all body weight loss groups were different from each other and that the stress level increased as the percentage of body weight loss increased. In terms of ergogenic aids according to body weight loss percentages, only the serious dehydration group was different from the minimal and significant dehydration groups, and this result shows that athletes in the serious dehydration group prefer laxatives, diet pills and/or diuretic pills to reduce more body weight. When the psychological effects were examined according to the percentage of body weight loss, it was observed that the serious dehydration group had a higher average than the minimal and significant dehydration groups, and as the percentage of body weight loss increased, the psychological effects would also increase (Table 4). Although the use of laxatives, diet, and/or diuretic pills is not a preferred method due to their inclusion on the WADA list, it has been reported that non-Olympic wrestlers occasionally prefer this method for RWL in local or non-doping controlled championships (Seker et al., 2024), it is not a recommended method because it will cause hypernatremic responses due to excessive water excretion. Although athletes know that the use of laxatives, diet, and/or diuretic pills is not a recommended method for losing weight, it is known that they resort to laxatives, diet, and/or diuretic pills as a faster solution instead of choosing excessive exercises, jogging with a raincoat and/or using sauna for the body weight they want to compete. However, it was also reported that as the amount of body weight loss increases, physiological and psychological stress in the body will also increase (Yildirim, 2015; Sariakcali et al., 2025), thus athletes will be exposed to psychological effects such as excessive irritability and fatigue, a decrease in their performance, and desire to do sports (Yagmur et al., 2019b; Yarar et al., 2019).

It was determined that there was a positive relationship between the weight loss-induced stress level and the percentage of body weight loss, as well as physiological and psychological effects (Table 5). This result shows that the increase in the percentage of body weight loss in wrestlers causes an increase in their stress levels and as their stress levels increase, their physiological and psychological effects will also increase. When the level of physiological and psychological effects of weight loss-induced stress on wrestlers was examined, it was determined that the stress that occurs during weight loss has an effect of 8.5% on the physiological effects of wrestlers and 25.8% on the psychological effects (Table 6). This result shows that wrestlers can be affected by other physiological factors at a level of 91.5% and by other psychological factors at a level of 74.2%.

As with every study, this study had some limitations. The results of this research consisted entirely of the results obtained through scales on the wrestlers’ feelings when losing weight before a competition. In this study, the main limitations were that the stress level was not determined through stress markers such as cortisol and the physiological and psychological effects were not determined through biochemical markers. This study was conducted only on wrestlers in the senior category from combat sports. Examining different combat sports such as judo, karate, taekwondo, boxing, and/or in different age groups may also provide different perspectives. Future studies are also needed to study the effects of body weight loss on competition performance in a formal competition environment. Thus, it may cause coaches to prevent their athletes from losing weight or to change their weight loss strategies. Finally, this study contained the results of a cross-sectional study. There is a need for more longitudinal studies in the literature on the effects of weight loss.

## 5 Conclusion

As a result, it was determined that as the percentage of body weight loss increases in wrestlers, the stress level will also increase, and as the stress level increases, the physiological and psychological effects will also increase. It is thought that weight loss-induced stress creates both physiological and psychological effects on wrestlers and may reduce their athletic performance. Therefore, manipulating the stress caused by weight loss and avoiding both physiological and psychological stressors will help minimize the effects on wrestlers during the weight loss process. Perhaps it will allow them to have a higher level of competition performance.

### 5.1 Suggestions

Of course, first of all, wrestlers should be prevented from RWL practice a short while before a competition. If weight loss practices cannot be prevented, gradual weight loss should be allowed and, if possible, should be controlled weekly by their coaches. It is important to prevent competitive athletes from continuing to experience RWL, which exposes them to physiological and psychological effects that disregard human health, by various changes to the competition rules by international federations. Moreover, wrestlers who are seriously dehydrated should not be included in the competition and they should be made to internalize that human health is more important than competition success.

## Data Availability

The raw data supporting the conclusions of this article will be made available by the authors, without undue reservation.
